# Mesenchymal stem cells in suppression or progression of hematologic malignancy: current status and challenges

**DOI:** 10.1038/s41375-018-0373-9

**Published:** 2019-01-31

**Authors:** Myoung Woo Lee, Somi Ryu, Dae Seong Kim, Ji Won Lee, Ki Woong Sung, Hong Hoe Koo, Keon Hee Yoo

**Affiliations:** 1 Department of Pediatrics, Samsung Medical Center, Sungkyunkwan University School of Medicine, Seoul, Korea; 2 Stem Cell & Regenerative Medicine Institute, Samsung Medical Center, Seoul, Korea; 3 CELLnLIFE Inc., Seoul, Korea; 4 Gyeongsang National University Hospital, Gyeongsang National University School of Medicine, Jinju, Korea; 5 Department of Health Sciences and Technology, SAIHST, Sungkyunkwan University, Seoul, Korea

**Keywords:** Stem-cell research, Stem-cell therapies, Preclinical research, Stem cells, Cell death

## Abstract

Mesenchymal stem cells (MSCs) are known for being multi-potent. However, they also possess anticancer properties, which has prompted efforts to adapt MSCs for anticancer therapies. However, MSCs have also been widely implicated in pathways that contribute to tumor growth. Numerous studies have been conducted to adapt MSCs for further clinical use; however, the results have been inconclusive, possibly due to the heterogeneity of MSC populations. Moreover, the conflicting roles of MSCs in tumor inhibition and tumor growth impede their adaptation for anticancer therapies. Antitumorigenic and protumorigenic properties of MSCs in hematologic malignancies are not as well established as they are for solid malignancies, and data comparing them are still limited. Herein the effect of MSCs on hematologic malignancies, such as leukemia and lymphoma, their mechanisms, sources of MSCs, and their effects on different types of cancer, have been discussed. This review describes how MSCs preserve both antitumorigenic and protumorigenic effects, as they tend to not only inhibit tumor growth by suppressing tumor cell proliferation but also promote tumor growth by suppressing tumor cell apoptosis. Thus clinical studies trying to adapt MSCs for anticancer therapies should consider that MSCs could actually promote hematologic cancer progression. It is necessary to take extreme care while developing MSC-based cell therapies in order to boost anticancer properties while eliminating tumor-favoring effects. This review emphasizes that research on the therapeutic applications of MSCs must consider that they exert both antitumorigenic and protumorigenic effects on hematologic malignancies.

## Introduction

Since the identification of mesenchymal stem cells (MSCs) from adult bone marrow (BM) [1], numerous studies have been performed globally to understand their characteristics and functions. Therefore, it is widely known that MSCs have multi-lineage potential, differentiating into various types of cells, such as adipocytes, chondroblasts, osteoblasts, and tissue macrophage-like cells [2, 3]. The multi-potent properties of MSCs make them promising therapeutic targets and one of the most indispensable sources of new clinical therapies [3]. In fact, MSCs have been widely used in regenerative medicine for bone and cardiovascular repair [4, 5]. Moreover, they can migrate to damaged tissue, can self-renew, and exert immunomodulatory and antitumor effects [5–7]. Despite extensive research carried out over the past 10 years, it is still unclear whether MSCs have tumor-suppressing or tumor-promoting effects [7].

MSCs have highly heterogeneous features [8]. This may explain why clinical trials involving MSCs have not developed beyond phase 1 and have been inconclusive [9]. Approximately 42 clinical trials investigating the role of MSCs on tumors are registered at www.clinicaltrials.gov, with only 13 targeting hematologic malignancies. These malignancies include myelodysplastic syndrome, leukemia, lymphoma, and multiple myeloma. Among the 13 clinical trials targeting hematologic malignancies, only one focused on anticancer effect of MSCs, while most of the other trials were related to the immunoregulatory effect of MSCs after stem cell transplantation. Further research is required to use MSCs for the treatment of hematologic malignancies. Here the various issues with safety, effectiveness, and the current status regarding the tumor-related effects of MSCs are presented.

## Dual role of MSCs in hematologic malignancy progression: MSCs suppress both proliferation and apoptosis

Cancer is difficult to target because it is not a single disease, but a class of diseases in which a group of cells display uncontrolled growth, invasion, and sometimes metastasis. Therefore, it seems impractical to develop one specific method to treat cancer. A variety of promising new therapies, such as cell therapy and immunomodulation, are being developed. Ongoing research suggests that MSCs are excellent targets for cell therapy in a variety of cancers. However, the reported antitumor effects are still controversial. Regardless of the type of cancer, some studies have shown inhibitory effects, while others demonstrate proliferative effects of MSCs on tumors [10]. For example, MSCs have tumoricidal effects on breast and lung cancer cell lines in vitro [11, 12] and on pancreatic tumors in vivo [13]. However, MSCs promote breast and melanoma cancer cell proliferation when co-cultured with tumor cell lines in vitro [14, 15]. They also increase tumor growth when injected into mice with lung or prostate cancer [16, 17]. Interestingly, both inhibitory and proliferative effects of MSCs have been reported in the same study [7, 12]. Several studies suggest that MSCs appear to influence pathways that can suppress both proliferation and apoptosis [18, 19]. The dual role of MSCs can be described as a “double-edged sword.” Therefore, it is important to understand its dual role in tumor cell proliferation.

There is less known about the function of MSCs in hematologic malignancies, such as leukemia, lymphoma, and multiple myeloma, than for solid malignancies, as described above. However, the dual function of MSCs may be applicable to hematologic cancers. According to several studies, it is evident that MSCs possess the ability to inhibit or promote tumor growth by suppressing proliferation or apoptosis of tumor cells, respectively, in hematologic malignancies [7, 10, 12]. Although minor reports have shown that MSCs can directly promote proliferation of hematologic malignant cells or promote apoptosis [20, 21], the primary hypothesis is that MSCs suppress both proliferation and apoptosis. Thus the use of MSCs for the treatment of hematologic malignancies is currently unclear, because inhibitory and promoting effects of MSCs on malignancies are known, both in vitro and in vivo [22, 23].

Moreover, mechanisms underlying antitumorigenic or protumorigenic effects remain unclear. Several different mechanisms have been suggested (Tables 1–4), some of which are favorable for the inhibition of hematologic malignancies. These include the possible use of MSCs as a delivery vehicle [24–26] to inhibit vascular growth [27–31] or to decrease cell proliferation by arresting the cell cycle [22, 23, 32–36]. These mechanisms favor the development of MSC-based therapies. However, MSCs are not favorable for clinical use because they have been implicated in supporting tumor vasculature [37–40], exerting immunomodulatory effects in cancer [41–43], and increasing the rate of metastasis and recurrence [44–53]. Moreover, recent studies have focused on how MSCs tend to protect tumor cells from drug-induced apoptosis, leading to chemo-resistance [54–62].

**Table 1:** Studies suggesting that MSCs inhibit hematologic malignancy by decreasing tumor cell proliferation in vitro

Isolated MSC	Tumor cell	Tumor cell no. (cells)	MSC:tumor cell ratio	Proposed mechanism	Reference
Mouse BM-MSC	Erythroleukemia (FBL3), ALL (P388), and B-lymphoma (A20)	2 × 10^4^	1:0.4, 1:1, 1:4, 1:10	Induction of cell cycle arrest and apoptosis of tumor cells	Song et al. [22]
Human BM-stromal cell line (HFCL)	AML (U937, HL-60, and HL-60/VCR)	2 × 10^4^	—	Induction of specific gene expression, leading to cell cycle blockage	Liang et al. [32]
Human BM-MSC	CML (BV173 and K562), AML (KG1a), and T-ALL (Jurkat)	5 × 10^3^	1:1, 1:5, 1:10, 1:100	Transient arrest of tumor cells in G1 phase	Ramasamy et al. [23]
Human BM-MSC	CML (K562 and BV173)	1 × 10^6^	1:10	—	Zhang et al. [54]
Human BM-MSC and CML patient’s BM-MSC	CML (K562 and patient’s cells)	—	1:10	Regulation of apoptosis-related protein expression and activation of the Wnt signaling pathway	Han et al. [71]
Human BM-MSC	CML (BV173) and T-ALL (Jurkat)	1 × 10^6^	1:5, 1:10, 1:50, 1:100	Induction of cell cycle arrest of leukemic cells	Sarmadi et al. [33]
Leukemia patient’s BM-MSC	CML (K562)	1 × 10^5^	1:10	Induction of cell cycle arrest of leukemic cells	Wei et al. [34]
Human UC-MSC	AML (HL-60) and CML (K562)	1 × 10^4^	1:1, 1:5, 1:10	Activation of p38 MAPK and induction of cell cycle arrest of leukemic cells	Tian et al. [35]
Human UC-MSC	CML (K562)	5 × 10^3^	MSC secretome used	Paracrine signaling by the secretome	Hendijani et al. [72]
Human AT-MSC	AML (HL-60) and CML (K562)	1 × 10^6^	1:10	Secretion of DKK-1 by NANOG	Zhu et al. [74]
Human BM-MSC	CML (patient’s cells)	1 × 10^4^	1:0.1, 1:1, 1:10	Production of IFN-ɑ	Zhang et al. [75]
Human UC-MSC	T-ALL (Jurkat)	2 × 10^6^	1:10	Activation of Notch signaling pathway	Yuan et al. [76]

*MSC* mesenchymal stem cell, *BM* bone marrow, *AML* acute myeloid leukemia, *CML* chronic myeloid leukemia, *UM* umbilical cord, *T-ALL* T cell acute lymphoblastic leukemia, *MAPK* mitogen-activated protein kinase, *IFN* interferon, *AT* adipose tissue

**Table 2:** Studies suggesting that MSCs affect hematologic malignancy by decreasing or increasing tumor growth in vivo

Isolated MSC	Tumor cell	Tumor cell no. (cells)	Animal type	Findings	Proposed mechanism	Reference
Mouse BM-MSC	B-lymphoma (A20)	1 × 10^4^	BALB/c mouse	Inhibit lymphoma cell growth	Inhibition of IL-10 secretion to immune evasion of lymphoma cells	Song et al. [22]
Human BM-MSC	Lymphoma (BJAB and SKW6.4)	2 × 10^6^	SCID mouse	Inhibit lymphoma cell growth	Induction of apoptosis of endothelial cells to form new blood vessels	Secchiero et al. [77]
Human AT-MSC	CML (K562)	2 × 10^5^	BALB/c-nu mouse	Inhibit leukemic cell proliferation	Induction of cell cycle arrest by secretion of DKK-1	Zhu et al. [74]
Human BM-MSC	CML (BV173)	1 × 10^6^	NOD/SCID mouse	Induce leukemic cell growth and reduce apoptosis	Formation of a cancer stem cell niche to preserve the self-renewal ability of leukemic cells	Ramasamy et al. [23]
Human AT-MSC	ALL (Reh, CCRF-CEM, SUP-T1, and CCRF-HSB2)	1 × 10^5^ 1 × 10^7^	NOD/SCID mouse	Induce leukemic cell growth	—	Lee et al. [110]

*MSC* mesenchymal stem cell, *BM* bone marrow, *CML* chronic myeloid leukemia, *ALL* acute lymphoblastic leukemia, *IL* interleukin, *AT* adipose tissue

**Table 3:** Studies suggesting that MSCs aggravate hematologic malignancy by suppressing tumor cell apoptosis in vitro

Isolated MSC	Tumor cell	Tumor cell no. (cells)	MSC:tumor cell ratio	Proposed mechanism	Reference
Human BM-MSC	B-ALL (patient’s cells)	1 × 10^6^	—	Secretion of soluble factors by MSCs	Manabe et al. [103]
Human BM-MSC	CLL (patient’s cells)	4 × 10^5^, 2 × 10^6^	—	Cell-to-cell contact of tumor cells with MSCs	Panayiotidis et al. [104]
Human BM-MSC	B-CLL (patient’s cells)	2 × 10^6^	—	Increased Bcl-2 expression by direct contact between leukemic cells and stromal cells	Lagneaux et al. [105]
Human BM-MSC	B-ALL (patient’s cells)	1 × 10^5^	1:10	Activation of Notch-3 and -4 signaling when tumor cells are in contact with MSCs	Nwabo Kamdje et al. [106]
Human stromal cell line (HS-5)	AML (patient’s cells)	4–6 × 10^5^	1:4~1:6	Direct cell-to-cell interactions regulating antiapoptotic effects, not including Bcl-2	Garrido et al. [107]
Mouse stromal cell line (MS-5)	AML (HL-60 and patient’s cells)	5 × 10^5^	—	Increased Bcl-2 expression	Konopleva et al. [108]
Human BM-MSC	BCP-ALL (patient’s cells)	—	—	Secretion of PGE_2_ from MSCs	Naderi et al. [109]
Leukemia patient’s BM-MSC	CML (K562)	1 × 10^5^	1:10	Activation of the PI3K-Akt-Bad pathway	Wei et al. [34]
Human UC-MSC	T-ALL (Jurkat)	2 × 10^6^	1:10	Activation of the Notch signaling pathway	Yuan et al. [76]
Human BM-MSC	CML (BV173)	1 × 10^6^	1:10	Transient cell cycle arrest conferring increased leukemic cell survival by preserving their proliferative ability	Ramasamy et al. [23]

*MSC* mesenchymal stem cell, *BM* bone marrow, *AML* acute myeloid leukemia, *CML* chronic myeloid leukemia, *UM* umbilical cord, *T-ALL* T cell acute lymphoblastic leukemia, *CLL* chronic lymphocytic leukemia, *PI3K* phosphoinositide 3-kinase, *PGE*_*2*_ prostaglandin E_2_

**Table 4:** Studies suggesting that MSCs induce drug resistance of hematologic malignant cells

Isolated MSC	Tumor cell	Drug	Proposed mechanism	Reference
Human BM-MSC	CML (K562 and BV173)	Imatinib	Upregulation of IL-7	Zhang et al. [54]
Human BM-MSC	CML (KBM-5)	Imatinib	Upregulation of CXCR4	Jin et al. [55]
Human BM-MSC	CML (BV173 and patient’s cells)	Imatinib	Upregulation of Bcl-xL expression and CXCL12/CXCR4 interaction	Vianello et al. [62]
Human BM-MSC and CML patient’s BM-MSC	CML (K562 and patient’s cells)	Adriamycin	Regulation of apoptosis-related protein expression and activation of the Wnt signaling pathway	Han et al. [71]
Human BM-MSC	AML (OCI-AML3)	Cytarabine	Regulation of leukemia–MSC interactions by ARC protein	Carter et al. [56]
Human BM-MSC	AML (U937 and KG1a)	Mitoxantrone	Upregulation of c-Myc	Xia et al. [57]
Human BM-MSC	AML (HL-60, THP1, U937, and patient’s cells)	Idarubicin	Activation of Notch signaling	Takam Kamga et al. [58]
Human BM-stromal cell line (HFCL)	AML (HL-60 and HL-60/VCR)	Topotecan	Upregulation of Bcl-2 expression	Liang et al. [32]
Mouse stromal cell line (MS-5)	AML (HL-60 and patient’s cells)	Cytarabine	Increased Bcl-2 and Bcl-xL expression	Konopleva et al. [108]
Human stromal cell line (HS-5)	AML (patient’s cells)	Cytarabine and Daunomycin	Direct cell-to-cell interactions	Garrido et al. [107]
Human BM-MSC and AML patient’s BM-MSC	AML (OCI-AML3 and patient’s cells) and pre-B ALL (Reh and RS4;11)	Cytarabine, Vincristine, and Doxorubicin	NF-κB activation in MSCs via a VCAM-1/VLA-4 axis	Jacamo et al. [137]
Human BM-MSC	T-ALL (Jurkat and patient’s cells)	Cytarabine and Methotrexate	Mitochondrial fission and p21 downregulation by activated ERK/Drp1	Cai et al. [59]
Human BM-MSC	pre-B ALL (Reh)	Genotoxic agents	Downregulation of p21 protein	Zhang et al. [60]
Human BM-MSC	T-ALL (Molt-4, Jurkat, CCRF-CEM, and CEM/C1)	Idarubicin	Activation of ERK by direct contact of leukemic cells and MSCs	Wu et al. [61]
Human UC-MSC	ALL (Jurkat)	Dexamethasone	Upregulation of Jagged 1 and overexpression of its receptor, Notch 1	Yuan et al. [76]

*MSC* mesenchymal stem cell, *BM* bone marrow, *AML* acute myeloid leukemia, *CML* chronic myeloid leukemia, *UM* umbilical cord, *T-ALL* T cell acute lymphoblastic leukemia, *CLL* chronic lymphocytic leukemia, *IL* interleukin, *ERK* extracellular signal–regulated kinase, *Drp1* dynamin-related protein 1, *CXCR* C-X-C chemokine receptor, *CXCL* C-X-C chemokine ligand, *ARC* apoptosis repressor with caspase recruitment domain, *NF* nuclear factor, *VCAM-1* vascular cell adhesion molecule-1, *VLA-4* very late antigen-4

## Antitumorigenic effects of MSCs

### Decreased proliferation of tumor cells in vitro

Although MSCs can inhibit and aggravate hematologic malignancies, it can also reduce proliferation of tumor cells in vitro. Studies demonstrating antitumor effects of MSCs and consequently inhibiting tumor growth are shown in Table 1. The mentioned studies utilized MSCs obtained from various sources. These sources include BM, which was the first source discovered for clinical applications, adipose tissue (AT), and umbilical cords (UC) [63, 64]. MSCs originating from these three sources are known to have similar phenotypes, surface antigen expression, and immunosuppressive properties [65, 66]. Our data also show that the antitumor effects of MSCs are not dependent on their origin. Most of the studies in Table 1 were carried out using leukemia cell lines, such as Jurkat, HL-60, and K562, instead of primary cells.

Another important consideration, besides the cell type used, is the concentration of the cells, specifically, the number of MSCs and tumor cells that were co-cultured. Culture conditions, especially the density of MSCs, is known to significantly affect morphology, proliferation rate, and secreted factors [67, 68]. Various types of studies, including gene expression profiles, have demonstrated the multi-functionality of MSCs, including immunoregulation, which can consequently alter the tumor-favoring or -suppressing effects of MSCs [69, 70]. Moreover, it has been suggested that antitumor effects observed in solid cancers are associated with a lower number of MSCs than those with tumor-promoting effects [7]. This association has not yet been suggested for hematologic malignancies but that may be due to lack of data. However, it still seems necessary to standardize the concentration of MSCs and hematologic malignant cells when they are co-cultured in vitro or injected into an in vivo model to safely and effectively use MSCs for further clinical adaptations.

There are many suggested mechanisms explaining the effects of MSCs on tumor cells; however; the most common and widely accepted mechanism is that MSCs induce tumor cell cycle arrest. Song et al. [22] co-cultured C57BL/6 mouse BM-derived MSCs with A20 murine B-lymphoma, FBL3 murine erythroleukemia, and P388 murine acute lymphoblastic leukemia (ALL) cells. They evaluated cell proliferation, apoptosis, cell cycle progression, and cytokine secretion. Consequently, MSCs suppressed the proliferation of lymphoma and leukemia cells in vitro via cell cycle arrest and reduced the levels of interleukin (IL)-10 secretion. Liang et al. [32] also suggested that cell cycle G0/G1 blockage, by transcriptional activation of specific genes, is the underlying mechanism of MSCs’ antitumor effect. In their study, the proliferation of acute myeloid leukemia (AML) cells co-cultured with a human BM fibroblastoid stromal cell line (HFCL) was inhibited. The percentage of G1 phase tumor cells, when co-cultured with HFCL, was significantly higher than that without HFCL and less S phase cells were observed. Similarly, Ramasamy et al. [23] found that MSCs downregulate cyclin D2 levels, leading to a transient cell cycle arrest of tumor cells in the G1 phase. MSCs were found to inhibit the self-renewal ability of cancer cells and their stromal environment could influence malignant diseases [54, 71]. Data presented by Sarmadi et al. [33] and Wei et al. [34] also support this finding, as they found significantly less proliferation of BV173/Jurkat and K562 cell lines when they were co-cultured with MSCs, due to tumor cell cycle arrest in the G0/G1 phase. They showed that proliferation was inhibited in a dose-dependent manner, mainly via cell-to-cell contact. Unlike these five reports mentioned above, Tian et al. [35] used MSCs derived from UC, and not from BM. However, their findings were similar, as proliferation was inhibited by MSCs due to the G0/G1 arrest. Moreover, in this study p38 mitogen-activated protein kinase (MAPK) was suggested as a potent cell proliferation and tumorigenesis suppressor of HL-60 and K562 cells. Gene silencing using small interfering RNA or pharmacological inhibition of p38 MAPK abrogated the inhibitory effect that lead to cell cycle arrest by MSCs. There are other reports suggesting different mechanisms for its antitumor effects besides cell cycle arrest. For example, Hendijani et al. [72] showed antitumor effects of the MSC secretome on leukemia cells. Thus certain secreted substances or paracrine signals may be involved, but the exact mechanism was not demonstrated in their report.

Shen et al. [73] showed that Wnt5a is a major modifier of tumor cell proliferation. When HL-60 leukemia cells were stimulated with the supernatant of adeno-Wnt5a MSCs, proliferation of leukemia cells highly reduced. Zhu et al. [74] have also emphasized the importance of the Wnt signaling pathway in regulating the antitumor effects of AT-derived MSCs, because of the increased secretion of Dickkopf-related protein (DKK)-1, a regulator of the Wnt signaling pathway. Another important underlying mechanism seems to be related to interferon (IFN)-α secretion [75]. Co-culturing MSCs with chronic myeloid leukemia (CML) mononuclear cells greatly inhibited their proliferation and this was associated with higher IFN-α levels in the supernatant of the co-cultured cells. IFN-α secretion increased with the increase in the concentration of MSC and co-culture duration. However, Yuan et al. [76] showed that Jurkat leukemia cell proliferation decreased owing to the UC-derived MSCs. Increased cellular expression of HES-1 transcription factor, which is involved in the Notch signaling pathway, was also observed in this study.

### Decreased tumor growth in vivo

Studies demonstrating the antitumor effects of MSCs by inhibiting tumor growth in vivo are shown in Table 2. After intravenous injection of MSCs into BALB/c mice with BALB/c-derived B-lymphoma A20 cells, Song et al. [22] showed a reduction in the incidence of lymphoma and improved survival rates. After co-culturing with MSCs, level of IL-10 in the supernatant of A20 cell cultures significantly decreased in a time-dependent manner. This could contribute to immune evasion. Furthermore, when co-cultured with MSCs, the fraction of A20 cells expressing intracellular IL-10 significantly increased, suggesting that MSCs inhibit the secretion of IL-10 by A20 cells. They concluded that the unexpected tumorigenic effect of MSCs shown in non-obese diabetic/severe combined immunodeficient mice with leukemia was a result of the animal type they used [23]. They mentioned that immunodeficient mice do not reflect the environment of autologous tumor development, and therefore, their in vivo data using BALB/c mice were more reliable. A study by Secchiero et al. [77] supports the tumor-suppressing effect of MSCs. Intraperitoneal injection of MSCs was performed 4 days after lymphoma cell injection. Tumor development was slower and was coupled with a large stromal infiltration and extensive intratumor necrosis. Moreover, when MSCs were directly co-cultured with endothelial cells, they observed a significant induction of endothelial cell apoptosis, suggesting that MSCs, under certain circumstances, may exert antiangiogenic activity. In addition, Zhu et al. [74] demonstrated that MSCs can inhibit K562 proliferation in vivo and that the inhibitory effect of MSCs was achieved through secretion of DKK-1, which suppresses the Wnt signaling pathway and inhibits cell proliferation.

### Favorable characteristics and mechanisms of MSCs for inhibition of hematologic malignancy

#### MSCs as delivery vehicles

MSCs are promising delivery vehicles and can be used for cancer therapy [24–26]. They are easily obtainable, hypo-immunogenic, rapidly expanded in vitro, and transplantable [78]. Moreover, MSCs are known to have inherent tumor-tropism capacities and thus can migrate to tumor sites. The cytotoxic effect of MSCs may be beneficial if they could migrate to tumor sites [79]. However, there have been a number of challenges in adapting the homing ability of MSCs for targeted delivery [80, 81]. Pharmacological properties of anticancer drugs improve with the use of drug delivery systems [81, 82]. Although there are limitations, including rapid clearance of nano-carriers from the bloodstream, a combination of the hypo-immunogenic and active targeting abilities of MSCs is promising for anticancer therapies [78]. MSCs can also be adapted as gene therapy carriers in a similar manner. They were first used to deliver IFN-β for treating ovarian cancer [83], reducing tumor growth, and prolonging survival in mouse models. Since then several researchers have used MSCs to deliver genes to certain tumors. Delivery of other factors, such as IFN-γ [84, 85], IL-12 [86, 87], IL-24 [88], and tumor necrosis factor-related apoptosis inducing ligand [89, 90], has also resulted in significant suppression of tumor cell growth.

Moreover, at the cellular and molecular level, MSCs produce most of their effects through paracrine action [91]. Extracellular vesicles (EVs), including exosomes and microvesicles, are lipid membrane-bound vesicles secreted from MSCs. EVs comprise a variety of molecules such as proteins, RNAs, and microRNAs that have originated from MSCs and these molecules are transferred to the other cells, such as cancer cells. Among the many subtypes of EVs, endosome-derived exosomes have emerged as physiologically relevant and powerful components of MSC secretome [92, 93]. Recent report showed that MSC secretome produced an antiproliferative effect on leukemic cells and a cytotoxic effect in combination with doxorubicin [72], indicating anti-leukemic potentials of exosome derived from MSCs. In addition, synthetically personalized exosome mimetics (EMs) could be the alternative vehicles for drug delivery as effective therapeutic agents. EMs from MSCs mixed with paclitaxel by extrusion could be isolated and drug-loaded MSC-EMs have revealed therapeutic efficiency against breast cancer [94]. MSC-EMs may be used as drug delivery vehicles for cancer treatment.

#### Inhibition of vascular growth

MSCs are known to have proangiogenic characteristics, resulting in tumor growth. However, there is evidence that MSCs can impair angiogenesis or vessel growth under certain conditions. They can migrate to endothelial cell-derived capillaries to produce reactive oxygen species [27, 28]. As a result, MSCs can activate endothelial cell apoptosis in vitro and suppress not only tumor growth but also capillary vessel density in a concentration-dependent manner in mouse melanoma models [28]. Reduced vascular density, leading to tumor growth inhibition, is known in various types of cancers, including breast cancer, glioma, and melanoma [29–31]. The underlying mechanisms appear to be involved in the modulation of the vascular endothelial cadherin/β-catenin signaling pathway [29] and downregulation of platelet-derived growth factor [30], IL-1β [30], and vascular endothelial growth factor (VEGF) [31]. Moreover, a recent study demonstrated that MSCs present in high numbers are potentially cytotoxic. Therefore, local injection of MSCs into tumor tissues may be an effective antiangiogenic treatment [28]. The inhibitory effect on tumor-related vessel growth has not been clearly demonstrated in hematologic cancers, but it may be an important mechanism, as these cancers are still dependent on vascular support [95].

#### Cell cycle arrest

Hematologic malignancies have fewer pathways compared to solid malignancies. The most common underlying process of tumor cell growth inhibition is cell cycle arrest, as listed in Table 1. Although DNA repair processes and cell cycle checkpoints seem to be linked to various cancers, induction mechanism of cancer cell arrest by antitumor agents, is still unknown. Since the precise molecular mechanisms of the cell cycle defects are not well understood, the effects of MSCs on leukemia or lymphoma are not well studied [36]. Several studies showing high level of cells arrested at G0/G1 phase did not reveal the underlying molecular processes. Therefore, further research is needed to study the mechanisms of tumor cell cycle arrest that consequently lead to the antitumor effects of MSCs on hematologic malignancies.

## Protumorigenic effects of MSCs

### Suppressed apoptosis of tumor cells in vitro

MSCs possess protumorigenic effects and suppress tumor cell apoptosis in vitro, as mentioned above. Studies emphasizing the tumor-favoring effect of MSCs are listed in Table 3. Most of the studies in Table 3 are based on MSCs derived from the BM. More than half of the studies shown in Table 3 used primary cancer cells obtained from leukemia patients instead of the reported cell lines [42–44.^96^–^100^], This is due to the difference between primary cancer cells and immortalized cell lines. Immortalized cell lines are known to have significant mutations, which can lead to altered cell traits, which could be a limitation while adapting them for clinical trials [101, 102]. Various factors and signaling pathways have been suggested to be involved in tumor-favoring mechanisms of MSCs. Cell-to-cell contact with MSCs seem to be critical for these factors and signaling pathways to be activated. The specific mechanisms are not fully understood; however, there are several studies emphasizing the importance of cell-to-cell contact. For example, Manabe et al. [103] demonstrated the antiapoptotic activity of MSCs in B-lineage ALL cells. Fifteen of the 18 B-lineage ALL cases showed 50% decrease in viability after 72 h of culture in medium alone, while apoptosis was prevented in 10 of the 12 ALL cases when they were cultured with allogeneic BM stromal cells as feeder layers. They suggested that certain soluble factors play an important role in the interaction between immature B cells and BM stroma cells. Panayiotidis et al. [104] showed that cells in 7 out of 10 cases of chronic lymphocytic leukemia (CLL) remained viable for a longer time when cultured with BM stromal cells. Here adherence of CLL cells to the BM stromal cell layers was also required for MSCs to protect cancer cells from apoptosis. Lagneaux et al. [105] demonstrated the dependence of apoptosis on direct contact between leukemic cells and stromal cells. They showed that adhesion of B-CLL cells to the stromal cells rescued them from apoptosis and extended their life span in vitro. Direct cell-to-cell contact was also found to be critical in a study by Nwabo Kamdje et al. [106]; however, they suggested that the antiapoptotic activity on leukemic cells is mediated by Notch-3 and Notch-4 or Jagged-1/-2 and Delta-like protein 1 in a synergistic manner, while many studies have failed to report specific mechanisms.

There are two other studies that used human and mouse stromal cell lines [107, 108] instead of primary MSCs, with similar results to the other studies in Table 3. Garrido et al. [107] cultured leukemic cells of 30 AML patients in direct contact with HS-5 human BM stromal cell monolayers or with HS-5 cells separated by transwell inserts. Leukemic cells were protected from culture- and drug-induced apoptosis when in direct contact. On the other hand, Konopleva et al. [108] used mouse stromal cell lines, which prevented apoptosis of HL-60 cells and primary AML blasts. They also observed increased B cell lymphoma-2 (Bcl-2) expression after co-cultivation of the leukemic cells with MSCs. Moreover, Naderi et al. [109] identified prostaglandin E_2_(PGE_2_) as a critical compound for antiapoptotic activity by showing that cell death is reversible upon inhibition of PGE_2_ synthesis. Primary B cell precursor ALL cells were protected from p53 accumulation and apoptosis through activation of cyclic adenosine monophosphate and protein kinase A signaling. Han et al. [71] explored the effects of MSC on proliferation, apoptosis, and secretion of cytokines during blastic phase-chronic myelogenous leukemia (CML-Bp). CML-Bp MSCs protected K562 CML cells and demonstrated an increased antiapoptotic ability, regulating the expression of apoptosis-related proteins and activating the Wnt pathway.

Some studies have demonstrated tumor-favoring effects as well as tumor-inhibiting effects, which are listed in both Tables 1 and 3 [23, 34, 76]. Apart from tumor cell cycle arrest as an explanation for antitumor effects of MSCs, phosphatidylinositol-3-kinase/protein kinase B (Akt)-Bad signaling [34] and Notch signaling [76] pathways have been implicated as the mechanisms that lead to antiapoptotic processes and tumor growth. Ramasamy et al. [23] showed that MSCs reduce apoptosis of BV173 CML cells. Their in vitro data showed that leukemic cells that had been in contact with MSCs were in a resting state (G0/G1), coupled with downregulation of cyclin D2. Such inhibition is likely to confer improved survival rates for leukemic cells by preserving their proliferative capacity and thus their self-renewal ability. Although the specific underlying processes and their interactions that mediate the effects of MSCs on hematologic malignancies are not yet clear, these reports support the idea of dual functionality of MSCs.

### Increased growth of tumors in vivo

Studies showing data regarding the in vivo tumor-favoring effects of MSCs on hematologic cancer are few. As shown in Table 2, Ramasamy et al. [23] suggested that MSCs present different characteristics based on the type of study, i.e., in vitro or in vivo. Specifically, MSCs were found to arrest leukemic cells in vitro, while tumor growth was aggravated when tumor cells were injected into mice. They suggested that MSCs have the ability to form a cancer stem cell niche, in which tumor cells contain the potential to proliferate and maintain malignant processes. Recently, we showed that MSCs facilitate the growth of ALL cells through the detection of viable luminescent ALL cells in an in vivo model [110]. This suggests that MSCs negatively affect hematologic malignancy such as recurrence of ALL cells. This should be considered before developing cell therapy products based on MSCs for the treatment of hematologic malignancy. Therefore, the dual function of MSCs and their effects on hematologic malignancies and solid cancers needs to be further studied before adapting them for clinical uses.

### Favorable characteristics and mechanisms of MSCs for aggravation of hematologic malignancy

#### Tumor vasculature support

Both hematologic malignancies and solid tumors require vascular support, which is promoted by MSCs [37–40.^111^–^117^]. MSCs are likely to support tumor vasculature directly by differentiating into pericytes or endothelial cells and indirectly by assisting the secretion of proangiogenic factors [15, 37–40]. Transplanted MSCs are engrafted into the perivascular niche when directly interacting with endothelial cells [118]. A population of MSC-like cells have been found in the perivasculature of mouse and human organs [119, 120]. Pericytes play an important role in vascular stabilization, but MSCs can also differentiate into endothelial cells, which may increase the density of vascularity and neovascularization [111, 112]. To support tumor vasculature, certain soluble factors must be secreted by MSCs. Vascular endothelial growth factor (VEGF) is well known as one of the proangiogenic factors involved in tumor angiogenesis [113, 114]. However, other proangiogenic cytokines are required for the angiogenic activity of VEGF. Recombinant VEGF alone does not show the same vascular support [115]. Other soluble factors, such as fibroblast growth factor-1, angiopoietin-1, and IL-6, are known to be secreted from MSCs [116, 117]. Several studies indicate that the vascular-supporting effect of MSCs is much more prominent than the inhibition of tumor capillary growth. Thus, if the angiogenic pathway can be blocked, developing MSC-based cell therapies that focus on the proangiogenic effects of MSCs may be promising.

#### Immunomodulatory effects of MSCs in cancer

There have been several studies emphasizing the immunoregulatory functions of MSCs, which can be adapted for clinical use. Although the main regulatory pathway remains unclear, MSCs have immunosuppressive properties, which may result in tumor growth in both solid cancers and hematologic malignancies. MSCs affect immunity via interactions with innate cellular components, like natural killer (NK) cells and adaptive cellular components, such as dendritic cells (DCs), B-lymphocytes, and T-lymphocytes [41–43]. MSCs can reduce the proliferative and cytotoxic ability of NK cells and can also inhibit maturation of DCs, which lead to the activation of T-lymphocytes. The involvement of various immunomodulatory factors is also known. These include transforming growth factor (TGF)-β [96, 121, 122], hepatocyte growth factor [96], indoleamine 2,3-dioxygenase with IFN-γ [97, 98], cyclooxygenase (COX)-1/-2 [99], PGE_2_ [99], inducible nitric oxide synthase [100], and A20 [123]. Some factors with immunomodulatory effects are known to aid MSC-induced tumor growth. TGF-β released by MSCs enhanced the epithelial–mesenchymal transition (EMT) of carcinoma, which is essential for tumor progression [121, 122]. Knockdown of A20 resulted in an antitumorigenic effect both in vitro and in vivo [123]. Moreover, multiple myeloma (MM)-MSC and CML-MSC-educated granulocytic-myeloid-derived suppressor cells showed an increase in immunomodulatory factors, such as arginase 1, tumor necrosis factor-ɑ, IL-1β, COX-2, and IL-6 [124, 125]. This supports an emerging concept regarding the contribution of MM-MSC and CML-MSC to tumor development and progression. However, function of other immunomodulatory factors remains unknown. Further research is needed to elucidate the role of immunomodulatory factors in tumor growth.

#### Metastasis and recurrence of malignancy

The most important process that contributes to the prometastatic effect of MSCs is the stimulation of EMT, a source of cancer-associated fibroblasts (CAFs) [44]. The EMT process develops more invasive phenotypes, resulting in local invasions and distant metastases [45–47]. It also affects the progression of various types of tumors, such as prostate cancer, pancreatic cancer, and breast cancer [48–50]. Administration of genetically labeled MSCs into mice with tumors significantly induced lung metastases [47].

There are other studies that identify specific molecules or pathways involved in these metastatic events. For example, chemokine (C-X-C motif) ligand (CXCL) type 16 secreted from prostate cancer and the subsequent CXCL16/C-X-C motif receptor (CXCR) type 6 signaling induces the conversion of MSCs into CAFs [48]. Moreover, promoting EMT through the Notch signaling pathway, by co-culturing with MSCs, also induces tumorigenesis [49]. Secretion of chemokine (C-C motif) ligand type 5 by MSCs was shown to be critical for metastasis in breast cancer [14], and MSCs support the entry of breast cancer into the BM through Tac1 regulation [50]. Entry into the BM suggest that the effect of MSCs on metastasis of solid cancers may be applicable to hematologic malignancies, as defects originate in the BM [50, 51].

Owing to an increase in cancer recurrence rate, the development of MSC-based anticancer therapies is considered. The failure of MSCs to be adapted into anticancer therapies is usually due to recurrence or relapse after the therapy, rather than a lack of primary response or initial remission [80]. Moreover, both metastasis and recurrence of malignancies are significantly related to tumor vasculature. MSCs migrate to the tumor parenchyma and differentiate into pericytes, inducing tumor vasculogenesis and promoting tumor recurrence [52]. Higher recurrence rates were also shown in patients with hematologic malignancies in a pilot clinical study [53]. There were two groups in the randomized clinical trial. Patients in one group received hematopoietic stem cells (HSCs) from a human leukocyte antigen-identical sibling donor, while the other group were co-transplanted with MSCs. Graft-versus-host disease was prevented when MSCs were co-transplanted with HSCs, but the relapse of hematologic malignancy was higher compared to the control group. Therefore, further research is required to adapt MSCs for clinical uses.

#### Enhancement of tumor cell stemness

MSCs are known to provide a favorable tumor-promoting microenvironment and increase tumor cell stemness [126–128]. MSCs are essential to the tumor-promoting microenvironment owing to their multilineage potential. They can differentiate into various types of tumor-related cells such as CAFs [128, 129]. Several studies have shown enhanced stemness of tumor-associated MSCs, which are integral components of the tumor microenvironment in various tumor cell lines [130]. Chosa et al. [131] introduced two novel mechanisms of enhanced stemness in MSCs: the scrapie responsive gene 1/BM stromal cell antigen-1 ligand–receptor combination and cell–cell adhesion through N-cadherin. On the other hand, MSCs have been shown to promote mammosphere formation partially via the epidermal growth factor (EGF)/EGF receptor/Akt pathway to regulate self-renewal through cytokine networks in breast cancer cells [132, 133]. Moreover, they regulate cancer stem cells via bone morphogenic protein signaling in ovarian cancer [134] and provide favorable tumor-promoting microenvironments through WNT/TGF-β signaling pathways in gastric carcinoma [135]. Besides their role in solid cancers, MSCs increase the stemness of cells in hematologic malignancies such as multiple myeloma via an activation of the Bruton tyrosine kinase signal pathway [136]. Thus MSCs play a critical role in tumor cell stemness in various types of tumors; however, the precise underlying mechanisms are still unclear. Therefore, to adapt MSCs for therapeutic use, further study is required to understand the enhancement of tumor cell stemness.

#### Drug resistance

Several studies demonstrating drug resistance of hematologic malignancies induced by MSCs are listed in Table 4, four of which showed CML cells becoming more resistant to chemotherapy. One of these studies used co-cultured primary CML cells to study the effect of the BM microenvironment in CML drug resistance [54]. They also found higher levels of IL-7 in the BM of CML patients in the blast crisis phase than healthy donors. IL-7 protects leukemic cells from imatinib-induced apoptosis via the Janus kinase 1/signal transducer and activator of transcription 5 pathway. KBM-5 CML cells were also protected from imatinib-induced cell death when they were co-cultured with MSCs [55]. Vianello et al. [62] showed that upregulation of CXCL12 and CXCR4 contribute to the drug-resistant ability of MSCs. In MSCs, differential expression of apoptosis-related proteins and activation of the Wnt pathway boost the antiapoptotic and drug-resistant activity [71].

Seven groups showed that MSCs induce chemo-resistance in AML. Carter et al. [56] emphasized the importance of an apoptosis repressor with caspase recruitment domain (ARC) protein. ARC induces the expression of IL-1β in AML cells. ARC mediates a complex regulatory circuit via nuclear factor (NF)-κB/IL-1β signaling in both AML cells and MSCs. This may be a novel target for AML, because this leads to the activation of numerous chemokine ligand/receptor axes that are closely associated with leukemic cell chemo-resistance. The gene c-Myc, which is involved in the regulation of various apoptotic molecules, may also play an important role, because their levels are upregulated in AML cells co-cultured with stroma [57]. Moreover, Notch inhibition abrogates stroma-induced chemo-resistance in AML, suggesting a potential therapeutic target for leukemia [58]. Expression of Bcl-2 may play an important role in resistance to topotecan [32] and cytarabine [108]. Irrespective of the pathways involved, direct cell-to-cell interaction with MSCs is required to make AML cells resistant to drugs [107]. Drug resistance of AML and ALL cells was due to NF-κB activation in MSCs via a vascular cell adhesion molecule-1/very late antigen-4 axis [137].

Extracellular signal–regulated kinase (ERK)/dynamin-related protein 1 (Drp1)-dependent mitochondrial fission and p21 downregulation are considered crucial for chemo-resistance in ALL. MSCs can alter mitochondrial dynamics induced by Drp1 activation, which can consequently protect leukemic cells from antitumor agents [59]. Downregulation of p21 may also explain the genotoxic agent-induced cell cycle arrest of ALL [60]. Wu et al. [61] used MSCs derived from BM and ALL cells, but the underlying mechanisms seemed different from the studies mentioned above. They have reported that ERK activation is important when ALL cells and MSCs are in direct contact. Yuan et al. [76] used MSCs from UC, instead of the BM, and the result was similar. However, they suggested different underlying mechanisms involving Notch signaling driven by Notch1 receptors, because significant upregulation of Jagged1 and overexpression of Notch1 were observed when Jurkat cells and UC-MSCs were co-cultured.

## Conclusions

MSC-based clinical outcomes have shown a wide range of variation likely due to non-standardized experimental methods, lack of specific cell surface markers to identify subsets of MSCs, and heterogeneous characteristics of MSCs that are easily affected by the surrounding environment. Therefore, further research is necessary to develop MSCs for cancer treatment. Moreover, there are many unclear and complicated aspects of cancer, especially hematologic malignancies, such as the tumor-related effects of MSCs. Several studies have been conducted to investigate the effects of MSCs in carcinogenesis or tumor microenvironments, but a single principle cannot explain both the antitumorigenic and protumorigenic functions of MSCs. Even though the underlying process remains unclear, the dual role of MSCs is widely acknowledged.

**Fig. 1:**
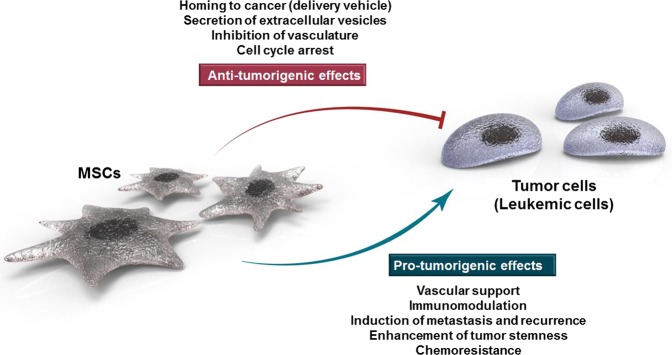
Scheme for the dual role of mesenchymal stem cells (MSCs) in hematologic malignancy. MSCs have both antitumorigenic and protumorigenic effects, as they tend to not only inhibit tumor growth by suppressing tumor cell proliferation but also promote tumor growth by suppressing tumor cell apoptosis

The antitumor effects of MSCs are mainly a result of suppressed proliferation of malignant cells. More specific mechanisms or molecules involved remain unclear, but arrest at the G0/G1 phase of the cell cycle is an acknowledged mechanism. To utilize this antitumorigenic activity for clinical use in the future, other factors must be considered. MSCs possess certain beneficial characteristics, such as the potential to be used as delivery vehicles and the ability to inhibit vascular growth and arrest the cell cycle (Fig. 1). However, unfavorable characteristics such as favoring tumor growth by suppressing apoptosis, supporting tumor vasculature, involvement in immunomodulation of cancer cells, activation of metastasis/recurrence, and protection of cancer cells from drug-induced apoptosis leading to chemo-resistance are a hindrance to their use as a therapeutic agent. Tumor-associated MSCs, essential components of the tumor microenvironment, are also associated with a protumorigenic effect because they tend to enhance tumor cell stemness (Fig. 1).

From the various underlying mechanisms that have been suggested and summarized here, it may be possible to develop MSC-based anticancer therapies by targeting individual pathways. Specifically, the development of molecules that can either increase antitumorigenic effects or decrease protumorigenic effects would be promising for advanced therapies. Detailed studies are required to overcome limitations such as the heterogeneous aspects of MSCs and the lack of standard study methods. Further research regarding the antitumor effects of MSCs should be conducted to develop safe and effective treatments for hematologic malignancies. Development of engineered or genetically modified MSCs may be a promising strategy, as they are safer and more efficient than the unstable and heterogeneous naive MSCs. As numerous researchers continue to overcome limitations and develop MSC-based cell therapies that target hematologic malignancies, there is hope that successful therapies will be developed. Until then, it is imperative that we approach MSC-based cell therapies with caution, considering the unfavorable outcomes described.
